# Impact of Maternal Folic Acid Supplementation on Descendants' Kidney in Adulthood

**DOI:** 10.1055/s-0043-1769001

**Published:** 2023-05-24

**Authors:** Thayane Ferreira da Costa Fernandes, Poliana dos Santos Conde, Flavia de Bittencourt Brasil, Marcos Roberto de Oliveira

**Affiliations:** 1 Universidade Federal do Rio de Janeiro, Macaé, RJ, Brazil; 2 Universidade Federal Fluminense, Rio das Ostras, RJ, Brazi; 3 Universidade Federal do Rio Grande do Sul, Porto Alegre, RS, Brazil

**Keywords:** Folic acid, Gestation, Kidney, Descendant, Adulthood, Ácido fólico, Gestação, Rim, Descendentes, Vida adulta

## Abstract

Supplementation with folic acid (FA) during gestation has been recommended by medical society all over the world, but some studies have shown that intake of high folic acid diet may unleash damages to the descendants. Objectives: Describing the effects of maternal supplementation with FA during gestation on offspring's kidney at late life stages. Data Source: It is a systematic review by which were consulted the following databases: Medline, through Pubmed, Lilacs, and SciELO. The research was performed using the keywords “Folic acid”, “Gestation” and “Kidney”. Study Selection: Eight studies were regarded for this systematic review. Data Collection: Only studies that evaluated folic acid consumption during gestation and its effects exclusively on descendants' kidney at several phases of life were regarded. Results: Gestational FA intake did not change the renal volume, glomerular filtration rate and the expression of some essential genes in the kidney of puppies whose dams were supplemented with FA. Maternal consumption of double FA plus selenium diet was effective in preserving antioxidant enzymes activity in the kidney of descendants from mothers exposed to alcohol. FA supplementation decreased some gross anomalies in the puppies caused by teratogenic drug despite of had not been effective in preventing some renal architectural damages. Conclusion: FA supplementation did not cause renal toxicity; it exerted an antioxidant protective effect and mitigated some renal disorders caused by severe aggressions.

## Resumo

A suplementação com ácido fólico (AF) durante a gestação tem sido recomendada pela sociedade médica em todo o mundo, mas alguns estudos têm mostrado que a ingestão de altas quantidades de ácido fólico na dieta pode desencadear danos aos descendentes. Objetivos: Descrever os efeitos da suplementação materna com AF durante a gestação no rim da prole em fases tardias da vida. Fonte de Dados: Trata-se de uma revisão sistemática realizada através da consulta das seguintes bases de dados: Medline, através da Plataforma Pubmed, Lilacs e Scielo. A pesquisa foi realizada utilizando-se as palavras-chave “Ácido Fólico”, “Gestação” e “Rim”. Seleção dos Estudos: Oito estudos foram considerados para esta revisão sistemática. Coleta de Dados: Foram incluídos estudos que abordaram o consumo de ácido fólico durante a gestação e seus efeitos exclusivamente no rim dos descendentes em diferentes fases da vida. Resultados: O consumo gestacional de AF não alterou o volume renal, a taxa de filtração glomerular e a expressão de alguns genes essenciais no rim dos filhotes de mães suplementadas com AF. A associação de AF e selênio na dieta materna foi eficaz na preservação da atividade de enzimas antioxidantes no rim da prole de mães expostas ao álcool. O consumo de AF diminuiu algumas anomalias importantes nos filhotes causadas por drogas teratogênicas, apesar de não ter sido eficiente na prevenção de alguns danos a arquitetura renal. Conclusão: A suplementação com AF não causou toxicicdade renal, exerceu efeito protetor antioxidante e mitigou algumas desordens renais causadas por agressões severas.

## Introduction

### Folic Acid Characterization

Folic acid (AF) is a water-soluble B (B9) vitamin, poorly stored in the body. The term “folic” comes from the Latin *folium* , leaf, due to its presence in leafy green vegetables such as spinach, cabbage, and broccoli besides viscera such as liver and kidney, milk, and egg. It is found in more than 90% as polyglutamates, which must be converted into monoglutamate before being absorbed. 1 FA is synthesized by microorganisms and higher plants, but not by mammals for which it is an essential nutrient needing to be ingested through food. 2 3 It can be also found in monoglutamate as a drug supplement, being quickly absorbed. 4 It has a pivotal role in purine and pyrimidine biosynthesis and consequently, in DNA and RNA formation. 5 It is also essential to specific metabolic reactions in the cell environment besides the growth and functioning of the organism. 1 FA works as coenzymes in the transport of simple carbon fragments and in the metabolism of amino acids. 6

### Absorption and Transport of Folic Acid

Polyglutamates of folates obtained from the diet are hydrolyzed into monoglutamate in the small intestine and absorbed by the intestinal mucosa. The enzyme named gamma-glutamyl hydrolase (γ-GH or glutamate carboxypeptidase II) is responsible for the hydrolysis of folylpolyglutamate and it is present on the villi of the small intestine epithelial cells characterized by brush shape. After hydrolysis, folate crosses basolateral membrane of intestinal mucosa cells, which contain their specific transporters, being released into the portal circulation. 7 FA is absorbed mainly in jejunum by passive transport, following a concentration gradient and by active transport when folate binds to reduced folate transporter 1 and 2 (RFT-1 and RFT-2) and folate binding protein (FBP). It can also be absorbed in the ileum just by passive transport. Folate is absorbed in a neutral pH environment (pH 7,4), with such a process being facilitated by the neutralization of gastric content by the alkaline pancreatic juice. The main form of circulating endogenous folate is 5-methyltetrahydrofolate, which is transported through plasm by low-affinity bindings with specific proteins, such as albumin and a soluble form of folate receptors (FR). 8 However, its concentrations are higher in red cells than in plasm due to its binding to hemoglobin. 9 Liver is able to absorb much of the folate from the portal circulation. The hepatic cells metabolize it into polyglutamate derivatives, retaining or releasing it in the blood or bile. 10 11 The folate excretion occurs mainly through bile in an approximate concentration of 100μg daily, 10 12 but it is reabsorbed in the small intestine. FA is filtered in the renal glomerulus and reabsorbed in the proximal contorted tubule. No FA is found in the urine, only its cleavage products. 13

### Gestation and Folic Acid

FA or B9 vitamin is spontaneously ingested in proper amounts by food in balanced diets. However, its deficiency becomes greater in women of childbearing age who intend to become pregnant, a period in which it is common to prescribe a drug supplement. 14 15 During gestation, the amount of FA ingested is insufficient to supply the daily needs that are increased in the pregnant. 16 Such vitamin has a fundamental role on cell proliferation, interfering in erythrocyte increasing, uterus enlargement, and development of both placenta and fetus, 17 becoming indispensable during pregnancy. According to Rangel-Rivera and Osma-Zambrano, 18 FA is essential for suitable formation and maintenance of several structures of the central nervous system, reducing the risk of severe language and attention disorders, schizophrenia, pre-eclampsia, low birth weight and premature birth. Meroanencephaly and spina bifida are the most common severe congenital malformations and both stem from defects of neural tube closure, can be prevented by FA intake in early gestation and immediately before such period. 19 Both periconceptional supplementation and during the first trimester of pregnancy has reduced the risk of recurrence of such defects in about 50 to 70%. 20 Due to this fact, the supplementation of pregnant with such nutrient has been recommended by medical societies all over the world both to prevent the first occurrence and the recurrence of those defects. 21 In China, for example, there was a significant decline in the number of congenital hydrocephalus cases after 2009, when it was applied a massive program of FA supplementation during gestation. 22 In Bangladesh also its prenatal intake decreased significantly the probability of myelomeningocele occurrence. 23 In Brazil, in 2002 the Ministry of Health regarded the folic acid as an essential medicine during prenatal care, recommending 400 µg (0,4mg) as daily dose 30 days before conception until the first trimester of gestation as a way of to prevent the occurrence of neural tube defects and maternal anemia. 8 This institution also recommends a dose of 5mg per day of FA for women who have congenital malformations history. 14 Nowadays, some studies have evaluated the impact of FA maternal intake besides those related to nervous system disorder prevention. Previously our group reported by systematic review that such supplementation during gestation exerted protective effects on liver of offspring in adulthood, 24 avoiding deleterious epigenetic changes and improving the cell defenses, especially in hostile maternal conditions, such as, alcohol exposition and deprivation of protein. Several studies agree about the importance of FA intake during gestation as a way of to prevent congenital malformations, but, some of them have questioned what would be the ideal doses and proposed that the ingestion of high quantities could trigger of some damages to descendants. Recent researches suggest that selective excessive intake of a type of vitamin can change negatively the metabolic activities and it is also applied to this supplementation. 25 Morakinyo et al., 26 for instance, demonstrated that high doses of FA during pregnancy or lactation decreased insulin sensitivity and adiponectin expression in offspring, predisposing to dyslipidemia and changes in glucose metabolism. Barua et al. 27 also report that FA high doses changes genomic function and affect the offspring's behaviour in mice. Leeming and Lucock 28 ponder that clinical and experimental studies are based on the fact such vitamin can prevent some malformations, but they do not consider the long-term effects, which can be deleterious. These authors suggest that high-dose supplementation can predispose to autism disorder and be associated to the increase in the number of children with such pathology. Other works corroborate it by reporting that ingestion throughout pregnancy may be associated with negative results in the development of the offspring's nervous system. 29 30

### Fetal Programming

Gestation is featured by physical and psychological changes, as result of body adaptations, 31 becoming the nutritional needs increased during such period. 16 Maternal nutritional state is pivotal to determine both metabolic and hormonal profile of descendants and stablish conditions which can remain throughout life. Waterland e Garza 32 defined this relationship as fetal programming, expression which associates nutritional changes in early life to diseases in adulthood, such as diabetes, obesity and arterial hypertension. Maternal nutrition is the main factor to determine intrauterine environment, due to its potential to change the expression of fetal genome and lead to development adaptations. Thus, the suitable nutrition may reduce the risks of developing chronic diseases in late life. 33 Several experimental models have been set up in order to evaluate the impact of maternal feeding on offspring development. Protein restriction, for example, is a widely used one and has been harmful to descendants inducing decrease in the number of nephrons in both adult male 34 and female rats 35 when it occurs during entire gestation. Exposure to prenatal undernutrition in human beings is also associated with premature brain aging in young adults. 36 In rats the protein shortage during gestation and lactation triggered some changes in male and female offspring's behaviour in a period equivalent to adolescence. According to the authors, both stereotyped behaviour and decreased social interaction observed can be associated with autism spectrum disorder. 37 Inappropriate amounts of micronutrients in the maternal diet can also cause some disorders in kidney development, such as both reduction in offspring's number of nephrons whose dams were submitted to vitamin A deprivatio 38 and renal glomeruli in descendants from dams that received iron deficient diet. 39 Regarding FA, some works have demonstrated that its deficiency during pregnancy changes cell division, which is more meaning in tissues with a high proliferation rate. 40 Cell multiplications, as well as rapid growth, which are central aspects of fetal development require a suitable folate supply. Meher et al. 41 found reduction of liver absolute weight in offspring from dams fed on low quantities of it during gestation and lactation, besides changed hepatic transcription factors expression. The hepatic protein levels involved with metabolism and neutralization of toxic products were also altered in male offspring from rats submitted to similar restriction during gestation. 42 Those changes remained until both six months and one year old, reinforcing the fetal programming concept. Due to some studies' question about the ideal quantity of FA that should be consumed during gestational period, this work intended to gather different experimental models and doses as well as its absence in order to report the impact in these situations. Besides it, few works have evaluated the effects of such supplementation in the kidney at late life. Because of its important role on homeostasis, this work goals to evaluate the supplementation effects exclusively during gestation in the offspring's kidney by current literature.

## Methods

It was performed research in the following databases: MedLine (Medical Literature Analysis and Retrieval System Online), through Pubmed, LILACS (Literatura Latino- Americana e do Caribe em Ciências da Saúde) and SciELO (Scientific Electronic Library Online). It was carried out through advanced research with the descriptors together: “folic acid”, “gestation” and “kidney” in English and Portuguese. In this way, we obtained 107 articles in the PubMed platform, two in LILACS, and none in Scielo. The word “offspring” was not included to enable a wide and comprehensive research. After preliminary reading, the articles that met the inclusion criteria were regarded: (1) studies with rats, mice, and human beings (2) studies that approached the effects of maternal folic acid consumption during gestation and lactation, and (3) studies that evaluated the offspring's kidney in several phases of life. The exclusion criteria were: (1) studies that performed FA supplementation in any other period than pregnancy and (2) articles that evaluated the effect on the mother.

## Results

The works regarded used different experimental models and evaluated several parameters. Thus, the results were separated into categories according to the evaluated criteria.

### Gestational Supplementation in Normal Conditions

#### Effects on Glomerular Filtration Rate

Lee et al. 43 followed children whose mothers received micronutrient supplementation during pregnancy and evaluated the long-term effects. These authors showed that maternal supplementation during early gestation was associated with a reduction of diastolic pressure in childhood despite the systolic pressure not being altered in those children. Also, the renal volume and glomerular filtration rate were not changed by such supplementation. Children whose mothers received high doses of iron (60mg) and folate (400 *μ* g) during gestation presented higher glomerular filtration rate when compared to offspring from supplemented mothers with half the quantity of iron. Lee et al. 43 performed a systematic review in order to comprehend the relationship between maternal nutrition and renal development exclusively in human beings, evaluating its structure and function in some nutritional situations. Among the works cited in such study, one of them reported a significantly lower risk to develop microalbuminuria in children six to eight years old whose mothers consumed FA during pregnancy. 44 Another study did not show any relation between such supplementation and change in the kidney of descendants at six years old. However, it suggested that higher maternal serum folate concentrations at early gestation were positively correlated with an increase in renal volume in childhood, but not with albuminuria risk. 45

#### Molecular Level Effect

One of the studies regarded in this manuscript analyzed both FA supplementation in specific organs and gestational period, investigating its impact at the first, second and third weeks singly or throughout pregnancy. 46 Folate plasmatic levels were higher in 30 to 42% in the pups from supplemented dams, meanwhile, its concentrations in the kidney and colon were not affected because of maternal intake. The intervention time did not cause difference about such parameters. Global DNA methylation was also observed in different organs. Relation to kidney, liver, and colon, was not observed any change in descendants whose dams received FA, regardless of supplementation time. The gene expression levels of essential genes for fetal development, such as α estrogen receptor (Er-α), glucocorticoid receptor (Gr), peroxisome proliferator-activated receptor alfa (Ppar-α), insulin-like growth factor 2 (Igf2) and peroxisome proliferator-activated receptor gamma (Ppar-γ) were not changed in the kidney of puppies from dams FA supplemented at any time of gestation. Similar results have been found in the brain and colon of those animals. Among the evaluated organs, only in the liver the expression of Er-α, Gr, Ppar-α genes was decreased in 15 to 25% in puppies whose mothers received FA in late gestation or throughout it. Based on results, the authors comment that the effects depend on the evaluated organ and the period in which it is applied.

### Gestational Supplementation in Hostile Conditions

#### Maternal Exposure to Alcohol

This study also selected articles that reported the impact of FA maternal intake associated with conditions which can predispose the descendants to diseases in long-term, such as, alcohol. Ojeda et al. 47 have investigated if such vitamin could reverse the damages from oxidative stress due to alcohol consumption during pregnancy and lactation in the offspring. They found out that the addition of FA and selenium (Se) on maternal diet mitigates the puppies' growth retardation, which is one of the harmful effects of alcohol exposure. The supplementation did not exert any effect on kidney relative weight; however, it prevented the reduction of protein content in renal tissues, found in those animals whose mothers consumed ethanol. The same puppies also had a decrease by 50% in creatinine clearance not improved by FA and Se. On the other hand, the supplemented diets were effective to preserve glutathione reductase enzyme activity in the kidney, which was decreased in descendants of dams exposed to alcohol. The double supplementation increased the superoxide dismutase (SOD) activity only in control animals and not in those whose mothers consumed alcohol. Catalase activity was preserved in the litter of dams that received Se and FA as well as. Another work published by the same authors reported that maternal alcohol intake during lactation caused reduction on litter body growth despite of did not alter the weight of any specific organ. Such disorder was reversed by double supplementation. Ethanol exposure also depleted Se in some organs as kidney, liver and brain, meanwhile the diet recovered this pattern. 48

#### Maternal Protein Restriction

Król et al. 49 have evaluate if FA combined with normal and hypoprotein diets during gestation could reverse the harmful effects from protein deprivation about minerals content in different tissues. Renal copper (Cu) content was reduced by maternal FA intake, but such levels were significantly lower in the offspring whose mothers received the vitamin associated with hypoprotein diet. Neither the protein-deficient nor FA supplemented diet affected iron (Fe) levels in the descendants' kidney. Maternal protein-defficient diet enriched by high quantity of FA (5mg) also was associated with higher renal levels of zinc (Zn). The gender was an important factor to determine Cu, Zn and Fe contents in the liver and kidneys, with female offspring having higher levels of such minerals than males.

#### Maternal Exposure to Other Drugs

Some studies have questioned if FA is able to prevent the disorders induced by genotoxic and teratogenic drugs during pregnancy. El-Ashmawy and Bayad 50 administered this vitamin together with azathioprine (AZA) between sixth and fifth days of gestation in rats and observed changes that happened to dams and fetuses. Despite not being the object of our study, some findings should be highlighted. Maternal weight gain, implantation sites and number of fetuses were close to control group. On the other hand, the dams which received only AZA presented higher number of dead fetuses and the living ones had marked reduction in body weight and growth, besides gross visceral and skeletal anomalies. The groups treated with FA displayed similar results to the control group, with significant decrease of those anomalies. The administration of FA and AZA during four weeks in a successive experiment became urea and creatinine serum levels close to the control group ( Chart 1 ). Otherwise, FA was not effective in reducing renal malondialdehyde (MDA) levels in those animals and preventing the architectural damages in the kidney, such as degeneration and tubular necrosis, swollen glomeruli, and infiltration of lymphocytes triggered by AZA. 50 51 52 53 54 55 56 57 58 59 60 61 62 63 64

**Chart 1:** General characteristics of the included studies

Author/Year	Sample	Intervention	Follow-up	Settings & participants	Objective	Assessment methods	Results
Lisle et al. (2003) 39	Control (153 mg Fe/kg diet, n 7) or low-Fe (3 mg/kg diet, n 6)	Experimental study	18 months	Offspring rats (n = 28)	Investigated the renal morphology of adult rats born to mothers who were Fe-deficient during pregnancy.	Kidney weight; Glomerular number and size; Systolic blood pressure	Maternal Fe restriction causes hypertension in the adult offspring that may be due, in part, to a deficit in nephron number.
Miliku et al. (2017) 45	Folic acid supplement (0.4–0.5 mg)	Population-based prospective cohort study from fetal life onwards	From pregnancy to 6 years of the child	Pregnant women and their (n = 4.226)	Examined the associations of folate, vitamin B12, and homocysteine concentrations during pregnancy with kidney outcomes in school-aged children.	Folate, vitamin B12 and homocysteine blood concentrations measured in early pregnancy and at birth (cord blood).	Folate, vitamin B12 and homocysteine concentrations during fetal life are associated with offspring kidney development.
Ly et al. (2016) 46	Folic acid supplement (2 mg and 5mg)	Experimental study	8 weeks	Sprague-Dawley Rats (n = 10)	This study evaluated whether maternal folic acid supplementation might change the offspring's.metabolism.	Brain, liver, kidney and colon of puppies were assessed about folate concentrations, global DNA methylation and expression. of Igf2, Er-α, Gr, Ppar-α e Ppar-γ genes.	Folic aid supplementation at late pregnancy or throughout gestation reduced the expression of Er-α, Gr and Ppar-α genes in the liver.
Ojeda et al. (2012) 47	(Se) (0.5 ppm) or with Se (0.5 ppm) + folic acid (8 ppm) administered to EtOH-exposed (20% v/v	Experimental study	8 weeks	Winstar rats (n = 6) female (n = 6) male	Diet supplemented with selenium or with Se + folic acid administered to EtOH-exposed dams during gestation and lactation prevents the oxidative EtOH-provoked effects in their offspring's kidneys.	Serum, urine and kidney, Se levels, creatinine clearance, antioxidant enzyme activities and lipid and protein peroxidation in the kidney.	Dietary supplementation improve renal development, Se deposits, and protein content while decreasing lipid and protein oxidation and modifying antioxidant enzymes' activity.
Ojeda et al. (2010) 48	Se (0.5 ppm) or with Se (0.5 ppm) plus folic acid (8 ppm) to ethanol-exposed (20% v/v)	Experimental study	8 weeks	Winstar rats (n = 6) female (n = 6) male	Supplemented diet with Se or with Se plus folic acid to ethanol-exposed dams prevents the ethanol-provoked effects in their offspring's Se deposits.	Selenium levels in the liver, kidney and testes.	Results show that ethanol decreases Se deposits in pups' heart, liver, kidney and testes. However Se levels in both pancreas and serum were increased by ethanol; it also compromised the weight and length of the offspring at the end of lactation.
Król et al. (2011) 49	(1) normal protein, normal folic acid (FA) diet (0.002-g FA); (2) protein-restricted, normal folic acid diet (0.002-g); (3) protein restricted, folic acid-supplemented diet (0.005 g FA); (4) normal protein, folic acid-supplemented diet (0.005 g FA).	Experimental study	6 weeks	Offspring (n = 48)	The aim of the study is investigate the influence of maternal diet during gestation on Fe, Zn, and Cu levels in the liver and kidney of adult rats.	The levels of Fe, Zn, and Cu in the livers and kidneys; Offspring's tissue mineral levels.	The results of this study show that maternal dietary folic acid and protein intake during pregnancy, as well as the type of postweaning diet, affect Fe, Zn and Cu levels in the offspring.
El-Ashmawy and Bayad (2016) 50	(1) AZA (25 mg/kg) (2) AZA, simultaneously with grape seed extract at the dose of 75 mg/kg (3) AZA, simultaneously with folic acid at the dose of 5 mg/kg;	Experimental study	4 weeks	Adult Wister rats (n = 40)	Investigate the influence of AZA on the fetal development and renal function and its co-administration with either folic acid (FA) or grape seed extract (GSE).	Kidney histology; Glutathione level (GSH); Lipid per oxidation content as malondialdehyde in the kidney tissue.	Maternal administrations of both FA and GSE protect against AZA-induced fetal malformations. Grape seed extract was more active than FA in potentiating the antioxidative defenses for controlling AZA-induced oxidative renal damages.
Hawkesworth et al. (2013) 64	(1) Fe30F: 30 mg iron and 400 µg of folate (2) Fe60F: 60 mg of iron and 400 µg of folate (3) MMS: Multiple micronutrient supplement	A trial follow-up study	Between November 2001 and October 2003 (recruited early in pregnancy) Between May 2007 and February 2009 when the descendants were 4.5 years old (children born)	Women were recruited early in pregnancy (n = 3.560) children (n = 3.267)	Assess the association between prenatal food and micronutrient supplementation and childhood blood pressure and kidney function. Women received either iron and folate or multiple micronutrient tablets daily.	Blood pressure; Kidney function.	Limited evidence for long-lasting impacts of pregnancy supplementation on offspring markers of kidney function.

## Discussion

The literature indicates that gestation is a critical period to determine the concept's future metabolic status and many factors can affect such development. In this review, we have found that FA maternal supplementation was not capable to change the DNA global methylation in the kidney, which seems to be beneficial, even though this result has not been observed in all organs. The supplementation performed throughout pregnancy is associated with such alteration in the brain and liver, with the first one being more susceptible. These organ-specific effects are probably related to the differences in both metabolism and folate demand in each of them. 51 The literature reports that changes in DNA methylation due to FA maternal intake vary according to the tissue, specific genes, interaction with other vitamins, among others.

Gestational supplementation did not change the expression of some genes, such as Igf2, Ppar-α e Ppar-γ in the kidney. Igf2 promotes fetal growth, 52 meanwhile Er-α is an estrogen nuclear receptor which allows the action of such hormone on reproductive development in embryo and fetus. 53 Ppar-α regulates lipid metabolism 54 and Ppar-γ regulates both glucose metabolism and storage of fatty acids. 55 The conservation of those genes expression is a relevant finding due to they are essential to several aspects of fetal development, such as growth and cell metabolism.

During embryogenesis, a new pattern of DNA methylation is set up 56 and is vulnerable to environmental factors such as maternal diet. Any alteration is likely to predispose disorders in long-term since aberrant or deregulated models of DNA methylation are associated with many diseases in human beings. 57

Organogenesis is a complex process that is under the influence of harmful conditions and drugs during the gestational period. FA seems to be effective to mitigate the disorders caused by teratogens or prevent some complications from such exposure in the kidney and other organs. Its protective effect observed in the offspring's kidney of dams exposed to AZA is corroborated by Ojeda et al. 58 when showing that FA administered with alcohol to pregnant rats avoided hepatic damages in the puppies at late life.

One of the ways to protection performed by FA is antioxidant activity. Its intake during pregnancy was able to preserve the glutathione reductase and catalase activity in the kidney of puppies whose mothers consumed alcohol. Catalase is related to superoxide dismutase in the removal of hydrogen peroxide and folate conjugated to catalase increases the ability of this enzyme to neutralize these reactive oxygen-derived species (RODS) being produced during some cell process that are potentially harmful. 59

Lipid and protein peroxidation is one of the damage mechanisms triggered by free radicals and RODS and occurs when there is imbalance between generation and capacity to eliminate it, which features oxidative stress. 60 The kidneys are susceptible organs due to plenty of polyunsaturated fatty acids in renal lipid composition 61 FA associated with selenium also reestablished the protein overall content in the kidney of puppies from dams exposed to ethanol, reinforcing its effectiveness in preventing the protein peroxidation caused by oxidative molecules.

According to Dennery, 62 the embryo development may be rather affected by such molecules due to the reduced capacity to neutralize them, since the embryo develops in an environment with relatively low oxygen levels. Oxidative stress can trigger off failure in embryo implantation, abortions, and congenital malformations. 60

Despite its protective properties, FA cannot be enough to suppress completely the impact of some acute and important aggressions during the gestational period, for instance, changes in renal ions transport unleashed by maternal deprivation of protein, 49 as well as the reduced glomerular filtration observed in the litter of ethanol-exposed dams during gestation was not prevented by FA + Se supplementation. 47 Hostile conditions can compromise embryonic nephrogenesis and in turn, alter the glomerular filtration rate. 63 Albeit FA has not reverted some disorders associated with nephrogenesis, it is suggested that folate deficiency might impact this process through epigenetic modulation. 43 There are hypotheses that little availability of folate, B12 vitamin, and other nutrients affects the volume of the kidneys and decreases the nephrons number of the offspring, predisposing to chronic renal disease in adulthood, 45 64 it reinforcing the concept of fetal programming.

In short, because of the large variety of maternal factors which may exert influence on fetal organogenesis and the intrinsic vulnerability of this process, studies that evaluate the supplementation with this one and other nutrients, as well as their absence, can enlighten the benefits and ensure the safe use in order to maintain the descendants' health.

## Conclusion

Gestational FA supplementation did not cause renal toxicity; it exerted antioxidant protective effect and mitigated some renal disorders unleashed by severe aggressions.

## References

[1] Vannucchi H Jordão Júnior A A Vitaminas hidrossolúveis São Paulo Sarvier 1998 191 207

[2] Green T Newton R Bourn D Estimated folic acid intakes from simulated fortification of the New Zealand food supply N Z Med J 2003 116(1168):U294 12607547

[3] Merrell B J McMurry J P Folic acid Treasure Island StatPearls 2020[cited 2022 Mar 12]. Available from:https://www.ncbi.nlm.nih.gov/books/NBK554487/

[4] Uehara S K Rosa G Associação da deficiência de ácido fólico com alterações patológicas e estratégias para sua prevenção: uma visão crítica Rev Nutr 2010 23 05 881 894

[5] Fakouri A Asghari A Akbari G Mortazavi P Effects of folic acid administration on testicular ischemia/reperfusion injury in rats Acta Cir Bras 2017 32 09 755 766 10.1590/s0102-865020170090000008 29019593

[6] Mahan L K Escott-Stump S Raymond J L Krause alimentos, nutrição e dietoterapia. 9a ed São Paulo Roca 1998

[7] McGuire J J Coward J K Pteroylpolyglutamates: biosynthesis, degradation, and function New York Wiley-Interscience 1984 135 190

[8] Baluz K Carmo M G Rosas G O papel do ácido fólico na prevenção e na terapêutica oncológica: revisão Rev Bras Cancerol 2002 48 04 597 60

[9] Lin Y Dueker S R Follett J R Quantitation of in vivo human folate metabolism Am J Clin Nutr 2004 80 03 680 691 10.1093/ajcn/80.3.680 15321809

[10] Whitehead V M Pharmacokinetics and physiological disposition of folate and its derivatives New York John Wiley & Sons 1986 177 205

[11] Horne D W Reed K A Hoefs J Said H M 5-Methyltetrahydrofolate transport in basolateral membrane vesicles from human liver Am J Clin Nutr 1993 58 01 80 84 10.1093/ajcn/58.1.80 8317394

[12] Suh J R Herbig A K Stover P J New perspectives on folate catabolism Annu Rev Nutr 2001 21 255 282 10.1146/annurev.nutr.21.1.255 11375437

[13] Williams W M Huang K C Renal tubular transport of folic acid and methotrexate in the monkey Am J Physiol 1982 242 05 F484 F490 10.1152/ajprenal.1982.242.5.F484 7081435

[14] Ministério da Saúde. Secretaria de Atenção à Saúde. Departamento de Atenção Básica Atenção ao pré-natal de baixo risco [Internet] Brasília, DF Editora do Ministério da Saúde 2012[cited 2022 Mar 20]. (Caderno de Atenção Básica; no. 32). Available from:http://bvsms.saude.gov.br/bvs/publicacoes/cadernos_atencao_basica_32_prenatal.pdf

[15] Moran V H A systematic review of dietary assessments of pregnant adolescents in industrialised countries Br J Nutr 2007 97 03 411 425 10.1017/S0007114507381373 17313700

[16] Hediger M L Scholl T O Khoo C S Fischer R L Diet, weight gain, and circulating micro-nutrients: evidence for nutritional depletion following adolescent pregnancy J Adolesc Health 1992 13 01 46

[17] McDonald S D Ferguson S Tam L Lougheed J Walker M C The prevention of congenital anomalies with periconceptional folic acid supplementation J Obstet Gynaecol Can 2003 25 02 115 121 10.1016/s1701-2163(16)30207-9 12577128

[18] Rangel-Rivera D A Osma-Zambrano S E Consumo de ácido fólico en el embarazo y reducción del riesgo de trastornos del espectro autista Med UIS. 2015 28 03 327 336 10.18273/revmed.v28n3-2015007

[19] Wald N J Morris J K Blakemore C Public health failure in the prevention of neural tube defects: time to abandon the tolerable upper intake level of folate Public Health Rev 2018 39 2 10.1186/s40985-018-0079-6 29450103 PMC5809909

[20] Santos L M Pereira M Z The effect of folic acid fortification on the reduction of neural tube defects Cad Saude Publica 2007 23 01 17 24 10.1590/S0102-311X2007000100003 17187100

[21] Valentin M Coste Mazeau P Zerah M Ceccaldi P F Benachi A Luton D Acid folic and pregnancy: A mandatory supplementation Ann Endocrinol (Paris) 2018 79 02 91 94 10.1016/j.ando.2017.10.001 29433770

[22] Liu J Jin L Li Z Prevalence and trend of isolated and complicated congenital hydrocephalus and preventive effect of folic acid in northern China, 2005-2015 Metab Brain Dis 2018 33 03 837 842 10.1007/s11011-017-0172-4 29388147

[23] Kancherla V Ibne Hasan M OS Hamid R Prenatal folic acid use associated with decreased risk of myelomeningocele: A case-control study offers further support for folic acid fortification in Bangladesh PLoS One 2017 12 11 e0188726 10.1371/journal.pone.0188726 29190654 PMC5708673

[24] Brasil F B Amarante L H Oliveira M R Maternal folic acid consumption during gestation and its long-term effects on offspring's liver: a systematic review Rev Bras Saúde Mater Infant 2017 17 01 17 25 10.1590/1806-93042017000100002

[25] Wiens D DeSoto M C Is high folic acid intake a risk factor for autism? - A review Brain Sci 2017 7 11 149 10.3390/brainsci7110149 29125540 PMC5704156

[26] Morakinyo A O Samuel T A Awobajo F O Oludare G O Mofolorunso A High-dose perinatal folic-acid supplementation alters insulin sensitivity in sprague-dawley rats and diminishes the expression of adiponectin J Diet Suppl 2019 16 01 14 26 10.1080/19390211.2018.1426076 29451831

[27] Barua S Kuizon S Brown W T Junaid M A DNA methylation profiling at single-base resolution reveals gestational folic acid supplementation influences the epigenome of mouse offspring cerebellum Front Neurosci 2016 10 168 10.3389/fnins.2016.00168 27199632 PMC4854024

[28] Leeming R J Lucock M Autism: Is there a folate connection? J Inherit Metab Dis 2009 32 03 400 402 10.1007/s10545-009-1093-0 19277892

[29] Raghavan R Fallin M D Wang X Maternal plasma folate, vitamin B12 levels and multivitamin supplementation during pregnancy and risk of autism spectrum disorder in the Boston Birth Cohort FASEB J 2016 30(S1):151.6

[30] INMA Project Valera-Gran D Navarrete-Muñoz E M Garcia de la Hera M Effect of maternal high dosages of folic acid supplements on neurocognitive development in children at 4-5 y of age: the prospective birth cohort Infancia y Medio Ambiente (INMA) study Am J Clin Nutr 2017 106 03 878 887 10.3945/ajcn.117.152769 28724645

[31] Costa M C Bezerra Filho J G Andrade Bezerra M G Veríssimo de Oliveira M I Carvalho de Oliveira R M De Vasconcelos Silva A R Gestação de risco: percepção e sentimentos das gestantes com amniorrexe prematura Enferm Glob 2010 9 03 1 11 10.6018/eglobal.9.3.110841

[32] Waterland R A Garza C Potential mechanisms of metabolic imprinting that lead to chronic disease Am J Clin Nutr 1999 69 02 179 197 10.1093/ajcn/69.2.179 9989679

[33] Wu G Bazer F W Cudd T A Meininger C J Spencer T E Maternal nutrition and fetal development J Nutr 2004 134 09 2169 2172 10.1093/jn/134.9.2169 15333699

[34] Woods L L Ingelfinger J R Nyengaard J R Rasch R Maternal protein restriction suppresses the newborn renin-angiotensin system and programs adult hypertension in rats Pediatr Res 2001 49 04 460 467 10.1203/00006450-200104000-00005 11264427

[35] Langley-Evans S C Welham S J Jackson A A Fetal exposure to a maternal low protein diet impairs nephrogenesis and promotes hypertension in the rat Life Sci 1999 64 11 965 974 10.1016/s0024-3205(99)00022-3 10201645

[36] Franke K Gaser C Roseboom T J Schwab M de Rooij S R Premature brain aging in humans exposed to maternal nutrient restriction during early gestation Neuroimage 2018 173 460 471 10.1016/j.neuroimage.2017.10.047 29074280

[37] Batista T H Giusti-Paiva A Vilela F C Maternal protein malnutrition induces autism-like symptoms in rat offspring Nutr Neurosci 2019 22 09 655 663 10.1080/1028415X.2018.1427660 29375017

[38] Lelièvre-Pégorier M Vilar J Ferrier M L Mild vitamin A deficiency leads to inborn nephron deficit in the rat Kidney Int 1998 54 05 1455 1462 10.1046/j.1523-1755.1998.00151.x 9844121

[39] Lisle S J Lewis R M Petry C J Ozanne S E Hales C N Forhead A J Effect of maternal iron restriction during pregnancy on renal morphology in the adult rat offspring Br J Nutr 2003 90 01 33 39 10.1079/bjn2003881 12844373

[40] Bunduki V Martinelli S Cabar F R Maternal and fetal serum and red blood cell folate levels in pregnancies complicated by neural tube defects Rev Bras Ginecol Obstet 1998 20 06 335 341 10.1590/S0100-72031998000600006Portuguese.

[41] Meher A Joshi A Joshi S Differential regulation of hepatic transcription factors in the Wistar rat offspring born to dams fed folic acid, vitamin B12 deficient diets and supplemented with omega-3 fatty acids PLoS One 2014 9 02 e90209 10.1371/journal.pone.0090209 24587285 PMC3938654

[42] Maloney C A Hay S M Reid M D A methyl-deficient diet fed to rats during the pre- and peri-conception periods of development modifies the hepatic proteome in the adult offspring Genes Nutr 2013 8 02 181 190 10.1007/s12263-012-0314-6 22907820 PMC3575887

[43] Lee Y Q Collins C E Gordon A Rae K M Pringle K G The relationship between maternal nutrition during pregnancy and offspring kidney structure and function in humans: a systematic review Nutrients 2018 10 02 E241 10.3390/nu10020241 PMC5852817 29466283

[44] Stewart C P Christian P Schulze K J Leclerq S C West K P Jr Khatry S K Antenatal micronutrient supplementation reduces metabolic syndrome in 6- to 8-year-old children in rural Nepal J Nutr 2009 139 08 1575 1581 10.3945/jn.109.106666 19549749

[45] Miliku K Mesu A Franco O H Hofman A Steegers E AP Jaddoe V WV Maternal and fetal folate, vitamin B12, and homocysteine concentrations and childhood kidney outcomes Am J Kidney Dis 2017 69 04 521 530 10.1053/j.ajkd.2016.11.014 28143670 PMC5408932

[46] Ly A Ishiguro L Kim D Maternal folic acid supplementation modulates DNA methylation and gene expression in the rat offspring in a gestation period-dependent and organ-specific manner J Nutr Biochem 2016 33 103 110 10.1016/j.jnutbio.2016.03.018 27152636

[47] Ojeda M L Nogales F Murillo M L Carreras O Selenium or selenium plus folic acid-supplemented diets ameliorate renal oxidation in ethanol-exposed pups Alcohol Clin Exp Res 2012 36 11 1863 1872 10.1111/j.1530-0277.2012.01788.x 22486362

[48] Ojeda M L Jotty K Nogales F Murillo M L Carreras O Selenium or selenium plus folic acid intake improves the detrimental effects of ethanol on pups' selenium balance Food Chem Toxicol 2010 48 12 3486 3491 20875836 10.1016/j.fct.2010.09.028

[49] Król E Krejpcio Z Chmurzynska A Folic acid and protein content in maternal diet and postnatal high-fat feeding affect the tissue levels of iron, zinc, and copper in the rat Biol Trace Elem Res 2011 144(1-3):885 893 10.1007/s12011-011-9048-3 21484405 PMC3241920

[50] El-Ashmawy I M Bayad A E Folic acid and grape seed extract prevent azathioprine-induced fetal malformations and renal toxicity in rats Phytother Res 2016 30 12 2027 2035 10.1002/ptr.5709 27561814

[51] Kim Y I Folate and carcinogenesis: evidence, mechanisms, and implications J Nutr Biochem 1999 10 02 66 88 10.1016/s0955-2863(98)00074-6 15539274

[52] Bergman D Halje M Nordin M Engström W Insulin-like growth factor 2 in development and disease: a mini-review Gerontology 2013 59 03 240 249 10.1159/000343995 23257688

[53] Bondesson M Hao R Lin C Y Williams C Gustafsson J A Estrogen receptor signaling during vertebrate development Biochim Biophys Acta 2015 1849 02 142 151 10.1016/j.bbagrm.2014.06.005 24954179 PMC4269570

[54] Grygiel-Górniak B Peroxisome proliferator-activated receptors and their ligands: nutritional and clinical implications–a review Nutr J 2014 13 17 10.1186/1475-2891-13-17 24524207 PMC3943808

[55] Michalik L Auwerx J Berger J P International Union of Pharmacology. LXI. Peroxisome proliferator-activated receptors Pharmacol Rev 2006 58 04 726 741 10.1124/pr.58.4.5 17132851

[56] Feng S Jacobsen S E Reik W Epigenetic reprogramming in plant and animal development Science 2010 330(6004):622 627 10.1126/science.1190614 21030646 PMC2989926

[57] Kulis M Esteller M DNA methylation and cancer Adv Genet 2010 70 27 56 10.1016/B978-0-12-380866-0.60002-2 20920744

[58] Ojeda M L Nogales F Jotty K Barrero M J Murillo M L Carreras O Dietary selenium plus folic acid as an antioxidant therapy for ethanol-exposed pups Birth Defects Res B Dev Reprod Toxicol 2009 86 06 490 495 10.1002/bdrb.20211 19918952

[59] Lee S Murthy N Targeted delivery of catalase and superoxide dismutase to macrophages using folate Biochem Biophys Res Commun 2007 360 01 275 279 10.1016/j.bbrc.2007.06.054 17586472

[60] Iborra A Palacio J R Martínez P Oxidative stress and autoimmune response in the infertile woman Chem Immunol Allergy 2005 88 150 162 10.1159/000087832 16129944

[61] Rodrigo R Rivera G Renal damage mediated by oxidative stress: a hypothesis of protective effects of red wine Free Radic Biol Med 2002 33 03 409 422 10.1016/s0891-5849(02)00908-5 12126763

[62] Dennery P A Oxidative stress in development: nature or nurture? Free Radic Biol Med 2010 49 07 1147 1151 10.1016/j.freeradbiomed.2010.07.011 20656021

[63] Araujo Guedes R C de Alburquerque Paiva A M Amâncio-dos-Santos A Vieira-Filho L D Oliveira da Paixão A D On some physiological aspects of ethanol repercussion on neural and cardiorenal functions Cent Nerv Syst Agents Med Chem 2009 9 04 277 288 10.2174/187152409789630433 20021360

[64] Hawkesworth S Wagatsuma Y Kahn A I Combined food and micronutrient supplements during pregnancy have limited impact on child blood pressure and kidney function in rural Bangladesh J Nutr 2013 143 05 728 734 10.3945/jn.112.168518 23514767 PMC3970319

